# Supplementary material for: Integration of Molecular Interactome and Targeted Interaction Analysis to Identify a COPD Disease Network Module
Source: Sci Rep. 2018 Sep 27;8:14439. doi: 10.1038/s41598-018-32173-z (PMC6160419; doi:10.1038/s41598-018-32173-z)
Supplement: Supplementary file 1 — Supplementary Material [file 41598_2018_32173_MOESM1_ESM.docx]

**Integration of Molecular Interactome and Targeted Interaction Analysis to Identify a COPD Disease Network Module**

Amitabh Sharma, Maksim Kitsak, Michael H. Cho, Asher Ameli, Xiaobo Zhou, Zhiqiang Jiang, James D. Crapo, Terri H. Beaty, Joerg Menche, Per S. Bakke, Marc Santolini, Edwin K. Silverman

**Supplementary Material**

**Supplementary table 1**: List of 163 genes in the expanded COPD disease network module.

**Supplementary table 2**: List of extracellular matrix organization pathway genes embedded in the COPD disease network module.

**Supplementary figure 1**: GWAS p-value distribution of COPD seed and DADA genes (from 100 to 500) and the rest of the genes in the human interactome.

**Supplementary figure 2**: Overlap between DADA and DIAMoND genes in the COPD network neighborhood by considering top 150 genes from both approaches.

**Supplementary figure 3:** **A**. Cumulative probability of having nodes with at least 96 neighbors in the interactome. **B**. Closeness of FAM13A interacting protein partners compared to random distribution.

**Supplementary figure 4:** Average degrees <k> of Cab, LR and all 96 partners of FAM13A. Significant difference in degree distribution was found between the Cab and LR method.

**Supplementary figure 5:** Fold change difference between COPD disease network module genes and non-module differentially expressed genes in eight COPD-specific gene-expression datasets without including seed genes.

**Supplementary figure 6: A**. COPD disease network module gene enrichment comparison with all genes in the gene expression data (p-cutoff=1). **B**. COPD disease network module gene enrichment by comparing with genes differentially expressed with p≤ 0.05.

**Supplementary figure 7:** COPD disease network module genes enrichment in eight different COPD-specific gene expression datasets. In each case, the two boxplots on the left side compare DE genes in the COPD disease network module (red) to DE genes outside of the module (gray), and the two boxplots on the right side compare a subset of 10 connected genes in the module (red) or in all DE genes (gray).

**Supplementary figure 8**: Histogram showing the expected number of genes differentially expressed (DE) in at least one dataset when choosing 9 random genes (gray). We find that the 9 Cab genes are all differentially expressed in at least one COPD-specific gene expression data set, which is statistically significant (Z=2.2 or p=0.016).

Acknowledgements for ECLIPSE :

ECLIPSE Investigators — Bulgaria: Y. Ivanov, Pleven; K. Kostov, Sofia. Canada: J. Bourbeau, Montreal; M. Fitzgerald, Vancouver, BC; P. Hernandez, Halifax, NS; K. Killian, Hamilton, ON; R. Levy, Vancouver, BC; F. Maltais, Montreal; D. O'Donnell, Kingston, ON. Czech Republic: J. Krepelka, Prague. Denmark: J. Vestbo, Hvidovre. The Netherlands: E. Wouters, Horn-Maastricht. New Zealand: D. Quinn, Wellington. Norway: P. Bakke, Bergen. Slovenia: M. Kosnik, Golnik. Spain: A. Agusti, J. Sauleda, P. de Mallorca. Ukraine: Y. Feschenko, V. Gavrisyuk, L. Yashina, Kiev; N. Monogarova, Donetsk. United Kingdom: P. Calverley, Liverpool; D. Lomas, Cambridge; W. MacNee, Edinburgh; D. Singh, Manchester; J. Wedzicha, London. United States: A. Anzueto, San Antonio, TX; S. Braman, Providence, RI; R. Casaburi, Torrance CA; B. Celli, Boston; G. Giessel, Richmond, VA; M. Gotfried, Phoenix, AZ; G. Greenwald, Rancho Mirage, CA; N. Hanania, Houston; D. Mahler, Lebanon, NH; B. Make, Denver; S. Rennard, Omaha, NE; C. Rochester, New Haven, CT; P. Scanlon, Rochester, MN; D. Schuller, Omaha, NE; F. Sciurba, Pittsburgh; A. Sharafkhaneh, Houston; T. Siler, St. Charles, MO; E. Silverman, Boston; A. Wanner, Miami; R. Wise, Baltimore; R. ZuWallack, Hartford, CT.

ECLIPSE Steering Committee: H. Coxson (Canada), C. Crim (GlaxoSmithKline, USA), L. Edwards (GlaxoSmithKline, USA), D. Lomas (UK), W. MacNee (UK), E. Silverman (USA), R. Tal Singer (Co-chair, GlaxoSmithKline, USA), J. Vestbo (Co-chair, Denmark), J. Yates (GlaxoSmithKline, USA).

ECLIPSE Scientific Committee: A. Agusti (Spain), P. Calverley (UK), B. Celli (USA), C. Crim (GlaxoSmithKline, USA), B. Miller (GlaxoSmithKline, USA), W. MacNee (Chair, UK), S. Rennard (USA), R. Tal-Singer (GlaxoSmithKline, USA), E. Wouters (The Netherlands), J. Yates (GlaxoSmithKline, USA).

**COPDGene^®^ Investigators – Core Units**

*Administrative Core*: James Crapo, MD (PI), Edwin Silverman, MD, PhD (PI), Barry Make, MD, Elizabeth Regan, MD, PhD

*Genetic Analysis Core*: Terri Beaty, PhD, Nan Laird, PhD, Christoph Lange, PhD, Michael Cho, MD, Stephanie Santorico, PhD, John Hokanson, MPH, PhD, Dawn DeMeo, MD, MPH, Nadia Hansel, MD, MPH, Craig Hersh, MD, MPH, Peter Castaldi, MD, MSc, Merry-Lynn McDonald, PhD, Emily Wan, MD, Megan Hardin, MD, Jacqueline Hetmanski, MS, Margaret Parker, MS, Marilyn Foreman, MD, Brian Hobbs, MD, Robert Busch, MD, Adel El-Bouiez, MD, Peter Castaldi, MD, Megan Hardin, MD, Dandi Qiao, PhD, Elizabeth Regan, MD, Eitan Halper-Stromberg, Ferdouse Begum, Sungho Won, Sharon Lutz, PhD

*Imaging Core*: David A Lynch, MB, Harvey O Coxson, PhD, MeiLan K Han, MD, MS, MD, Eric A Hoffman, PhD, Stephen Humphries MS, Francine L Jacobson, MD, Philip F Judy, PhD, Ella A Kazerooni, MD, John D Newell, Jr., MD, Elizabeth Regan, MD, James C Ross, PhD, Raul San Jose Estepar, PhD, Berend C Stoel, PhD, Juerg Tschirren, PhD, Eva van Rikxoort, PhD, Bram van Ginneken, PhD, George Washko, MD, Carla G Wilson, MS, Mustafa Al Qaisi, MD, Teresa Gray, Alex Kluiber, Tanya Mann, Jered Sieren, Douglas Stinson, Joyce Schroeder, MD, Edwin Van Beek, MD, PhD

*PFT QA Core, Salt Lake City, UT*: Robert Jensen, PhD

*Data Coordinating Center and Biostatistics*, *National Jewish Health, Denver, CO*: Douglas Everett, PhD, Anna Faino, MS, Matt Strand, PhD, Carla Wilson, MS

*Epidemiology Core*, *University of Colorado Anschutz Medical Campus, Aurora, CO*: John E. Hokanson, MPH, PhD, Gregory Kinney, MPH, PhD, Sharon Lutz, PhD, Kendra Young PhD, Katherine Pratte, MSPH, Lindsey Duca, MS

**COPDGene^®^ Investigators – Clinical Centers**

*Ann Arbor VA:* Jeffrey L. Curtis, MD, Carlos H. Martinez, MD, MPH, Perry G. Pernicano, MD

*Baylor College of Medicine, Houston, TX*: Nicola Hanania, MD, MS, Philip Alapat, MD, Venkata Bandi, MD, Mustafa Atik, MD, Aladin Boriek, PhD, Kalpatha Guntupalli, MD, Elizabeth Guy, MD, Amit Parulekar, MD, Arun Nachiappan, MD

*Brigham and Women’s Hospital, Boston, MA*: Dawn DeMeo, MD, MPH, Craig Hersh, MD, MPH, George Washko, MD, Francine Jacobson, MD, MPH

*Columbia University, New York, NY*: R. Graham Barr, MD, DrPH, Byron Thomashow, MD, John Austin, MD, Belinda D’Souza, MD, Gregory D.N. Pearson, MD, Anna Rozenshtein, MD, MPH, FACR

*Duke University Medical Center, Durham, NC*: Neil MacIntyre, Jr., MD, Lacey Washington, MD, H. Page McAdams, MD

*Health Partners Research Foundation, Minneapolis, MN*: Charlene McEvoy, MD, MPH, Joseph Tashjian, MD

*Johns Hopkins University, Baltimore, MD*: Robert Wise, MD, Nadia Hansel, MD, MPH, Robert Brown, MD, Karen Horton, MD, Nirupama Putcha, MD, MHS,

*Los Angeles Biomedical Research Institute at Harbor UCLA Medical Center, Torrance, CA*: Richard Casaburi, PhD, MD, Alessandra Adami, PhD, Janos Porszasz, MD, PhD, Hans Fischer, MD, PhD, Matthew Budoff, MD, Harry Rossiter, PhD

*Michael E. DeBakey VAMC, Houston*, TX: Amir Sharafkhaneh, MD, PhD, Charlie Lan, DO

*Minneapolis VA:* Christine Wendt, MD, Brian Bell, MD

*Morehouse School of Medicine, Atlanta, GA*: Marilyn Foreman, MD, MS, Gloria Westney, MD, MS, Eugene Berkowitz, MD, PhD

*National Jewish Health, Denver, CO*: Russell Bowler, MD, PhD, David Lynch, MD

*Reliant Medical Group, Worcester, MA*: Richard Rosiello, MD, David Pace, MD

*Temple University, Philadelphia, PA:* Gerard Criner, MD, David Ciccolella, MD, Francis Cordova, MD, Chandra Dass, MD, Gilbert D’Alonzo, DO, Parag Desai, MD, Michael Jacobs, PharmD, Steven Kelsen, MD, PhD, Victor Kim, MD, A. James Mamary, MD, Nathaniel Marchetti, DO, Aditi Satti, MD, Kartik Shenoy, MD, Robert M. Steiner, MD, Alex Swift, MD, Irene Swift, MD, Maria Elena Vega-Sanchez, MD

*University of Alabama, Birmingham, AL:* Mark Dransfield, MD, William Bailey, MD, J. Michael Wells, MD, Surya Bhatt, MD, Hrudaya Nath, MD

*University of California, San Diego, CA*: Joe Ramsdell, MD, Paul Friedman, MD, Xavier Soler, MD, PhD, Andrew Yen, MD

*University of Iowa, Iowa City, IA*: Alejandro Cornellas, MD, John Newell, Jr., MD, Brad Thompson, MD

*University of Michigan, Ann Arbor, MI*: MeiLan Han, MD, Ella Kazerooni, MD, Carlos Martinez, MD

*University of Minnesota, Minneapolis, MN*: Joanne Billings, MD, Tadashi Allen, MD

*University of Pittsburgh, Pittsburgh, PA*: Frank Sciurba, MD, Divay Chandra, MD, MSc, Joel Weissfeld, MD, MPH, Carl Fuhrman, MD, Jessica Bon, MD

*University of Texas Health Science Center at San Antonio, San Antonio, TX*: Antonio Anzueto, MD, Sandra Adams, MD, Diego Maselli-Caceres, MD, Mario E. Ruiz, MD

**Supplementary Figure 1:**

**Supplementary Figure 2:**

**Supplementary Figure 3:**

**Supplementary Figure 4:**

**Supplementary Figure 5:**

**Supplementary Figure 6:**

**Supplementary Figure 7:**

**Supplementary Figure 8:**
